# Metabolic reprogramming in hepatocellular carcinoma: mechanisms of immune evasion and therapeutic implications

**DOI:** 10.3389/fimmu.2025.1592837

**Published:** 2025-04-30

**Authors:** Bocheng Gao, Yan Lu, Xingyue Lai, Xi Xu, Shuhua Gou, Zhida Yang, Yanju Gong, Hong Yang

**Affiliations:** ^1^ School of Medical and Life Sciences, Chengdu University of Traditional Chinese Medicine, Chengdu, China; ^2^ School of Basic Medical Sciences, Chengdu University of Traditional Chinese Medicine, Chengdu, China

**Keywords:** hepatocellular carcinoma, metabolic reprogramming, immune evasion, tumor microenvironment, TME, tumor-associated macrophages (TAMs), immunotherapy

## Abstract

Hepatocellular carcinoma (HCC) is a leading cause of cancer-related deaths worldwide, with limited treatment options for advanced stages. Metabolic reprogramming is a hallmark of cancer, enabling tumor cells to adapt to the harsh tumor microenvironment (TME) and evade immune surveillance. This review involves the role of metabolic reprogramming in HCC, focusing on the dysregulation of glucose, lipid, and amino acid metabolism, and its impact on immune evasion. Key metabolic pathways, such as the Warburg effect, fatty acid synthesis, and glutaminolysis, are discussed, along with their influence on tumor-associated macrophages (TAMs) and immune cell function. Targeting these metabolic alterations presents a promising therapeutic approach to enhance immunotherapy efficacy and improve HCC patient outcomes.

## 1 Introduction

Hepatocellular carcinoma (HCC) demonstrates limited therapeutic responsiveness, with objective response rates remaining at approximately 20% in clinical settings (1, 2). While immunotherapy has emerged as a potential therapeutic strategy for advanced HCC (3), its clinical efficacy remains constrained by tumor immune evasion mechanisms operating within the immunosuppressive tumor microenvironment (TME) (4–6). Metabolic reprogramming drives both tumorigenesis and disease progression through profound alterations in core metabolic pathways including glycolysis, fatty acid synthesis, and glutamine metabolism (7–9). These adaptations not only fulfill the biosynthetic and bioenergetic demands of rapidly proliferating tumor cells but also actively shape an immunosuppressive TME. Critically, the metabolic crosstalk between malignant cells and immune cell populations within the TME facilitates immune evasion mechanisms and confers resistance to immunotherapeutic interventions (10). The liver’s immunosuppressive nature and the TME’s enrichment with regulatory T cells (Tregs), myeloid-derived suppressor cells (MDSCs), and TAMs drive HCC progression and limit immunotherapy efficacy (11, 12).

During HCC development, malignant cells continuously adapt their metabolic patterns to acquire sufficient nutrients for self-renewal and proliferation in the hypoxic and nutrient-deprived TME (13). These metabolic alterations not only induce phenotypic and functional changes in TAMs but also lead to their metabolic reprogramming, enabling them to exert immunosuppressive functions and promote tumor progression and metastasis (14). Recent advances in HCC metabolism research have provided significant insights. However, current immunotherapies, including checkpoint inhibitors targeting the PD-1/PD-L1 or CTLA-4 pathways, face substantial challenges in HCC due to the profoundly immunosuppressive metabolic landscape (15). This review explores metabolic reprogramming in HCC, focusing on dysregulated enzymes and pathways in glucose, lipid, amino acid, and nucleotide metabolism, and discusses how effectively targeting dysregulated metabolic pathways can synergize with existing immunotherapies, thereby potentially overcoming immune evasion and improving clinical outcomes.

## 2 Glucose metabolism in HCC

### 2.1 Glucose metabolic reprogramming: key enzymes and pathways

Glucose metabolic reprogramming is one of cancer cells’ most prominent metabolic features. HCC cells upregulate the expression of glucose transporters 1 and 2 (GLUT1 and GLUT2) to increase glucose uptake, and the downregulation of GLUT1 and GLUT2 significantly inhibits HCC cell growth and proliferation (16, 17). Once inside the cell, glucose is phosphorylated by hexokinase (HK) to form glucose-6-phosphate (G6P). Among these, HK2 is overexpressed in various cancers, including HCC, and its high expression is associated with poor prognosis in HCC patients (18). Inhibition of HK2 expression has been shown to enhance the therapeutic efficacy of sorafenib in HCC (19). M1-like macrophages consume large amounts of glucose for glycolysis, upregulating anabolic pathways to provide substrates and rapid energy production. Studies have demonstrated that the glycolysis inhibitor 2-deoxy-D-glucose (2-DG) significantly reduces ATP levels in HCC M1-like macrophages, inhibiting glycolysis (20). Additionally, glycolysis fuels the pentose phosphate pathway (PPP), generating NADPH to produce reactive oxygen species (ROS), which is crucial for the phagocytic activity of M1-like macrophages (21). Another key enzyme in glycolysis, pyruvate kinase (PK), catalyzes the conversion of phosphoenolpyruvate to pyruvate. PK has two isoforms, PKM1 and PKM2, with PKM2 being highly expressed in HCC and associated with poor patient prognosis (22). Finally, pyruvate is converted to lactate by lactate dehydrogenase (LDH) (23), and elevated LDH levels in HCC tissues and plasma are significantly correlated with poor prognosis (24).

The upregulation of key glycolytic enzymes in HCC cells is closely linked to the activation of oncogenes and pro-tumor signaling pathways. Hypoxia-inducible factor 1α (HIF-1α) plays a critical role in regulating glycolysis in HCC cells by transcriptionally upregulating several glycolytic enzymes, including GLUT1 and HK2 (25). HIF-1α is a key transcription factor that enables cells to adapt to low oxygen levels and is essential for glycolysis in TAMs. Studies have shown that LPS induces the phosphorylation of PKM2, promoting the formation of a nuclear PKM2/HIF-1α complex that binds to the HIF-1α promoter, driving its expression and enhancing glycolysis in TAMs in HCC (26). Inhibition of the phosphoinositide 3-kinase/protein kinase B/mammalian target of rapamycin (PI3K/Akt/mTOR) signaling pathway plays a significant role in tumorigenesis and progression (27). The PI3K/Akt/mTOR signaling pathway, which plays a significant role in tumor progression, has also been implicated in promoting glycolysis in HCC cells by regulating GLUT4 and HK2 (28, 29).

### 2.2 Impact of glucose metabolism on immune evasion

The inefficient delivery of nutrients and oxygen, along with the poor clearance of metabolic waste in tumors, creates a hypoxic and acidic TME, which significantly impairs anti-tumor immune responses (30–32). HCC cells enhance glycolysis, producing large amounts of lactate, which acidifies the TME and suppresses immune cell function. In HCC patients, serum HIF-1α levels are negatively correlated with prognosis, as HIF-1α recruits macrophages to hypoxic regions of the TME via chemokines such as CCL-2 and endothelin (31, 33). Accumulated HIF-1α promotes the release of IL-1β by TAMs through the TLR4/TRIF/NF-κB signaling pathway, facilitating immune evasion (34). Tumor cells enhance lactate dehydrogenase A (LDH-A) activity to convert pyruvate into lactate, which is then exported out of the cell via monocarboxylate transporter 4 (MCT4), further acidifying the TME. This acidic environment inhibits the anti-tumor functions of cytotoxic T lymphocytes, natural killer (NK) cells, and dendritic cells (DCs) while promoting the immunosuppressive functions of Tregs and MDSCs (35).

Lactate also plays a pivotal role in stabilizing HIF-1α, which in turn upregulates the expression of arginase-1 and VEGF. These factors drive the polarization of macrophages toward the M2 phenotype, an alteration that promotes tumor growth and metastasis (36). The M2 macrophages, characterized by their pro-tumorigenic properties, further secrete factors that enhance angiogenesis and tissue remodeling, creating a feedback loop that perpetuates tumor progression and immune suppression (37). In summary, the hypoxic and acidic conditions within the HCC TME, driven by enhanced glycolysis and lactate production, create a hostile environment for anti-tumor immunity. This environment not only impairs the function of key immune cells but also promotes the recruitment and polarization of immunosuppressive cells, thereby facilitating tumor immune evasion and progression.

Previous evidence underscores the direct immunomodulatory role of the pyruvate kinase M2 (PKM2)/HIF-1α axis in macrophages, T cells, and NK cells. PKM2 can translocate to the nucleus and serve as a coactivator for HIF-1α, driving transcription of glycolytic enzymes and immunosuppressive mediators (38, 39). This heightened glycolytic state not only fuels tumor metabolism but also skews macrophage polarization toward an M2-like phenotype, suppresses T cell effector function through local lactate accumulation, and hampers NK cell cytotoxicity (39, 40). Consequently, interventions targeting PKM2’s nuclear function or inhibiting the PKM2/HIF-1α complex can disrupt key immunosuppressive circuits in the TME and restore anti-tumor immune responses. In addition to tumor cells, non-tumor stromal cells also undergo glycolytic shifts that contribute to this immunosuppressive milieu. For example, cancer-associated fibroblasts (CAFs) may increase glucose consumption and release lactate, fueling adjacent HCC cells while simultaneously creating an acidic TME that hampers T cell and NK cell function (41). This reciprocal metabolic interplay between tumor and stromal cells magnifies immune evasion by shaping an environment that favors HCC survival and progression.

## 3 Dysregulation and immune modulation functions of lipid metabolism in HCC

### 3.1 Lipid metabolic reprogramming in HCC

Lipid metabolic reprogramming in HCC is characterized by enhanced fatty acid uptake and *de novo* synthesis, increased cholesterol synthesis, and reduced fatty acid oxidation. These alterations are closely associated with HCC development and progression. HCC cells promote growth and proliferation by increasing the uptake of exogenous fatty acids (42). CD36, a fatty acid transporter, facilitates the uptake of long-chain fatty acids (LCFAs) and oxidized low-density lipoprotein (Ox-LDL), playing a crucial role in lipid metabolism and serving as a significant tumor marker (43). Studies have shown that tumor cells utilize CD36 on their cell surface to uptake fatty acids, and CD36 is overexpressed in HCC cells. CD36 promotes HCC progression by activating Wnt and TGF-β signaling pathways and inducing epithelial-mesenchymal transition (EMT) (44–46). These findings suggest that CD36 may be a novel target for enhancing HCC immunotherapy through metabolic pathways.

*De novo* fatty acid synthesis (*de novo* FAS) is a critical pathway for tumor cells to acquire lipids. HCC cells exhibit heightened *de novo* FAS activity (47). Key enzymes involved in *de novo* FAS, such as ATP citrate lyase (ACLY) and fatty acid synthase (FASN), are overexpressed in HCC, and their upregulation is associated with poor prognosis (48). Several transcription factors and signaling pathways regulate *de novo* FAS in HCC cells. Sorafenib disrupts monounsaturated fatty acid synthesis mediated by stearoyl-CoA desaturase-1 (SCD1) through the ATP-AMPK-mTOR-SREBP1 signaling pathway, leading to HCC cell death (49). Dysregulated cholesterol synthesis is another hallmark of lipid metabolic reprogramming in HCC. HMG-CoA reductase (HMGCR), the rate-limiting enzyme in cholesterol biosynthesis, is upregulated in HCC and is the target of statins, which regulate plasma cholesterol levels (50). Increased mitochondrial cholesterol content in HCC cells reduces mitochondrial membrane permeability, inhibiting cytochrome c release and conferring resistance to chemotherapy (50). Recent studies have highlighted the importance of fatty acid β-oxidation (FAO) in tumor progression. SIRT4, a member of the Sirtuin family, functions as an ADP-ribosyl transferase and regulates FAO and mitochondrial gene expression in liver and muscle cells (51). Loss of SIRT4 enhances the expression of FAO-related genes such as pyruvate dehydrogenase kinase 4 (PDK4) and carnitine palmitoyl transferase 1 (CPT1), as well as mitochondrial genes such as cytochrome c (CytC) and isocitrate dehydrogenase 3α (IDH3α) in TAMs. SIRT4 deficiency also promotes M2 polarization of TAMs through the PPARδ-STAT3 signaling pathway, driving HCC progression (52).

### 3.2 Role of lipid metabolism in immune evasion and tumor progression

Liver fatty acid-binding protein (L-FABP) is highly expressed in HCC tissues and has been shown to regulate lipid metabolism and inflammation in host cells (53). TAMs derived from HCC highly express L-FABP and promote NK cell recruitment through the production of IFN-β, mediating anti-tumor effects (54). However, TAMs also produce prostaglandin E2 (PGE2), which suppresses anti-tumor immunity. In HCC, TAMs upregulate cyclooxygenase-2 (COX2) and prostaglandin E synthase 1 (PGES1) to produce high levels of PGE2, which inhibits IFN-γ production and NK cell cytotoxicity (55). HCC cells enhance FAO, promoting M2 polarization of TAMs and suppressing immune cell function (56). The FAO inhibitor etomoxir blocks FAO activity in TAMs, inhibiting their pro-tumor functions (57, 58). Consequently, targeting FAO in TAMs may not only impede tumor progression but also enhance the efficacy of immune checkpoint inhibitors and other immunotherapies by restoring anti-tumor immune responses within the TME (59, 60).

## 4 Amino acid metabolism and immune suppression in HCC

### 4.1 Amino acid metabolic reprogramming

Amino acid metabolism in HCC is characterized by increased glutaminolysis (61). The alanine-serine-cysteine transporter 2 (ASCT2) is the primary transporter of glutamine in cells (62). ASCT2 is overexpressed in HCC and is associated with poor prognosis (63). Glutamine serves as a major energy source for HCC cells, being catabolized by glutaminase (GLS) into glutamate, which enters the tricarboxylic acid (TCA) cycle to generate energy. Glutamine synthetase (GS) converts ammonia and glutamate into glutamine. Targeting GS with small-molecule inhibitors can reprogram TAMs into antigen-presenting cells, exerting anti-tumor effects. GLS has two isoforms, GLS1 and GLS2. GLS1 promotes tumor cell growth in various cancers (64), while GLS2 suppresses cancer cell proliferation and migration (65). In HCC, GLS1 is overexpressed and promotes cell proliferation via the AKT/GSK3β/CyclinD1 pathway (66). High GLS1 expression is positively correlated with stemness in HCC cells, and targeting GLS1 reduces stemness by increasing mitochondrial ROS and suppressing the Wnt/β-catenin pathway (67). In contrast, GLS2 is downregulated in HCC and inhibits tumor growth by negatively regulating the PI3K/AKT signaling pathway (68–71). However, despite these well-established roles, there are also contradictory or context-dependent findings concerning GLS1 and GLS2 in HCC (72). These conflicting observations underscore the complexity of glutamine metabolism in HCC and highlight the importance of studying tumor-stage specific expression and activity of these isoforms. Further investigation into the interplay between GLS1/GLS2 and other metabolic or signaling pathways will be crucial for designing precision therapies targeting glutaminolysis in HCC.

M2-like TAMs highly express arginase-1, which hydrolyzes arginine into ornithine and urea. Ornithine serves as a precursor for polyamines and collagen, contributing to extracellular matrix formation and tissue repair. Ornithine is further metabolized by ornithine decarboxylase into polyamines, promoting M2 polarization of macrophages and tumor cell growth (73). Additionally, serine metabolism plays a crucial role in HCC progression. Studies have confirmed that under conditions of glucose or glutamine starvation, the serine biosynthesis pathway in HCC cells is significantly enhanced. c-Myc promotes the production of glutathione, progression of the cell cycle, and synthesis of nucleic acids by upregulating the expression of various SSP enzymes, thereby facilitating the survival and proliferation of HCC cells (74–76).

### 4.2 Amino acid metabolism influences immune cell function in HCC

Amino acid metabolism in HCC significantly influences the immune response of various cells, playing a crucial role in tumor progression. TAMs exhibit increased activity of indoleamine 2,3-dioxygenase 1 (IDO1), which converts tryptophan into kynurenine. Kynurenine induces T cell death, reduces the number of pro-inflammatory T lymphocytes, and diminishes T cell anti-tumor activity (77). CHEN et al. (78) demonstrated that early-activated CD69^+^ T cells enhance IDO activity in TAMs, accelerating tryptophan metabolism. This metabolic shift not only promotes T cell proliferation and cytokine production but also activates Tregs, thereby facilitating HCC cell proliferation and metastasis. Excessive arginine consumption in HCC depletes arginine levels in the TME, impairing NK cell proliferation and IFN-γ production (79, 80). *In vitro* studies have shown that low arginine levels in the HCC microenvironment suppress the expression of NK cell activation receptors such as NKp46 and NKp30 (81, 82). Additionally, the acidic microenvironment resulting from high lactate levels inhibits NK cell cytotoxicity and cytokine production (83). Given the crucial role of amino acids in T cell activation and effector function, reducing IDO1-mediated tryptophan depletion or alleviating excessive arginine consumption could reverse T cell dysfunction and synergize with immunotherapies such as anti-PD-1/PD-L1 antibodies. Indeed, clinical trials evaluating IDO1 inhibitors in combination with checkpoint inhibitors in other cancer types suggest potential avenues for analogous therapeutic approaches in HCC (84).

Collectively, the interplay between metabolic reprogramming (glucose, lipid, and amino acid metabolism) and immune evasion is a pivotal driver of HCC progression. As summarized in **Table 1**, dysregulated enzymes and pathways in these metabolic networks not only sustain tumor cell proliferation but also actively suppress anti-tumor immunity by polarizing TAMs and acidifying the TME. Future combination therapies that simultaneously target metabolic checkpoints and enhance T cell functionality may thus open new pathways toward reversing immunosuppression and improving HCC patient outcomes.

**Table 1:** Key metabolic pathways in HCC and their roles in immune evasion.

Metabolic Pathway	Key Enzymes/Transporters	Regulatory Mechanisms	Impact on Immune Evasion
Glucose Metabolism	GLUT1/2, HK2, PKM2, LDH-A	HIF-1α, PI3K/Akt/mTOR signaling	Acidic TME (via lactate) inhibits CTLs/NK cells; M2 polarization of TAMs via HIF-1α/IL-1β
Lipid Metabolism	CD36, FASN, ACLY, SCD1	Wnt/TGF-β signaling, SREBP1	FAO promotes M2 TAMs; PGE2 suppresses NK cells; L-FABP recruits NK cells
Amino Acid Metabolism	ASCT2, GLS1, IDO1, Arg-1	AKT/GSK3β/CyclinD1, TLR4/NF-κB	Tryptophan depletion by IDO1 induces T cell death; arginine depletion impairs NK function
Cholesterol Metabolism	HMGCR, SIRT4	PPARδ-STAT3 signaling	Mitochondrial cholesterol confers chemotherapy resistance

## 5 Therapeutic targeting of metabolic pathways in HCC

Given the dysregulation of key enzymes and signaling pathways in glucose, lipid, amino acid, and nucleotide metabolism in HCC, targeting these metabolic abnormalities represents a promising therapeutic strategy. Aspirin has been shown to inhibit HCC by targeting the overexpression of GLUT1, reducing glucose uptake in HCC cells (85). Similarly, HK2 can be targeted by resveratrol (86), miRNAs (87, 88), and Ras-associated glycolysis inhibitors (89). Celastrol, a triterpenoid compound derived from the medicinal plant *Tripterygium wilfordii*, covalently modifies glycolytic enzymes like HK2 in M1 macrophages, shifting their metabolism from glycolysis to oxidative phosphorylation and promoting M2 polarization, thereby alleviating lipid accumulation, inflammation, and fibrosis in the liver (90). Additionally, the SCD1 inhibitor SSI-4, when combined with sorafenib, enhances anti-tumor efficacy, suggesting a novel therapeutic strategy for HCC (91).

Beyond targeting metabolic enzymes, dysregulated metabolic pathways in HCC are also potential therapeutic targets. TAM metabolism influences the expression of PD-L1 and PD-1, which mediate immune suppression. Clinical studies have shown that targeting the PD-L1/PD-1 pathway significantly improves clinical outcomes in HCC patients (92). In HCC, PD-L1 is primarily produced by PD-L1^+^ macrophages (93). Inflammatory factors are pivotal in diseases’ progression (94–97). A recent study found that fibronectin 1 derived from HCC interacts with Toll-like receptor 4, activating glycolysis in macrophages via the PKM2/HIF-α signaling pathway and promoting the secretion of pro-inflammatory cytokines IL-1β and TNF-α, which enhance PD-L1 expression on macrophages, ultimately leading to immune evasion (98). These findings highlight the intricate link between metabolic reprogramming and immunosuppression in HCC, suggesting that interventions aimed at inhibiting glucose uptake, fatty acid synthesis, or glutaminolysis could potentiate the efficacy of immune checkpoint inhibitors. As combination therapies that pair PD-1/PD-L1 or CTLA-4 blockade with metabolic modulators continue to be explored, a personalized approach to targeting both tumor metabolism and immune evasion may emerge as a new standard for advanced HCC (**Table 2**).

**Table 2:** Therapeutic strategies targeting HCC metabolism.

Target	Drug/Intervention	Mechanism	Stage (Preclinical/Clinical)
Glucose Transport	Aspirin	Inhibits GLUT1, reduces glucose uptake	Preclinical
Glycolysis	2-DG, Celastrol	Inhibits HK2/LDHA, shifts TAM metabolism	Preclinical
Fatty Acid Synthesis	Sorafenib + SSI-4	Inhibits SCD1, induces cell death	Preclinical
Glutaminolysis	GLS1 inhibitors (e.g., CB-839)	Reduces glutamine dependency, suppresses stemness	Clinical trials
PD-L1/PD-1 Pathway	Atezolizumab + Bevacizumab	Blocks immune checkpoint, enhances T cell activity	FDA-approved (HCC)
IDO1	Epacadostat	Restores tryptophan, reverses T cell suppression	Phase II trials

## 6 Conclusion

Metabolic reprogramming, encompassing the dysregulation of glucose, lipid, and amino acid metabolism, directly fosters immune evasion and immunotherapy resistance in HCC. By creating a microenvironment characterized by hypoxia, nutrient depletion, and acidification, these metabolic changes blunt the activity of cytotoxic T cells, NK cells, and other effector populations while promoting the expansion of immunosuppressive cell subsets such as Tregs and M2-polarized TAMs. This reciprocal relationship between aberrant metabolism and immunosuppression underlies one of the most formidable challenges to effective HCC treatment, as it contributes to the suboptimal response rates to current immunotherapies. Targeting metabolic pathways offers promising therapeutic strategies to overcome immune evasion and improve HCC treatment outcomes. Future research should focus on developing metabolic inhibitors and combining them with existing immunotherapies to enhance their efficacy. In particular, early-phase clinical trials investigating combination regimens that integrate metabolic interference with anti-PD-1/PD-L1 therapies are poised to illuminate the feasibility and potential synergy of dual-targeting approaches in HCC.

## References

[1] Zeng L Su J Qiu W Jin X Qiu Y Yu W. Survival outcomes and safety of programmed cell death/programmed cell death ligand 1 inhibitors for unresectable hepatocellular carcinoma: result from phase III trials. Cancer Control. (2022) 29:10732748221092924. doi: 10.1177/10732748221092924 35418272 PMC9014721

[2] Zhai X Xia Z Du G Zhang X Xia T Ma D. LRP1B suppresses HCC progression through the NCSTN/PI3K/AKT signaling axis and affects doxorubicin resistance. Genes Dis. (2023) 10:2082–96. doi: 10.1016/j.gendis.2022.10.021 PMC10363646 37492741

[3] Liang R Hong W Zhang Y Ma D Li J Shi Y. Deep dissection of stemness-related hierarchies in hepatocellular carcinoma. J Transl Med. (2023) 21:631. doi: 10.1186/s12967-023-04425-8 37717019 PMC10505333

[4] Xia Z Chen S He M Li B Deng Y Yi L. Editorial: Targeting metabolism to activate T cells and enhance the efficacy of checkpoint blockade immunotherapy in solid tumors. Front Immunol. (2023) 14:1247178. doi: 10.3389/fimmu.2023.1247178 37575246 PMC10415066

[5] Zhang P Pei S Wu L Xia Z Wang Q Huang X. Integrating multiple machine learning methods to construct glutamine metabolism-related signatures in lung adenocarcinoma. Front Endocrinol (Lausanne). (2023) 14:1196372. doi: 10.3389/fendo.2023.1196372 37265698 PMC10229769

[6] Zhang X Zhuge J Liu J Xia Z Wang H Gao Q. et al. Prognostic signatures of sphingolipids: Understanding the immune landscape and predictive role in immunotherapy response and outcomes of hepatocellular carcinoma. Front Immunol. (2023) 14:1153423. doi: 10.3389/fimmu.2023.1153423 37006285 PMC10063861

[7] Soltani M Zhao Y Xia Z Ganjalikhani Hakemi M Bazhin AV. The importance of cellular metabolic pathways in pathogenesis and selective treatments of hematological Malignancies. Front Oncol. (2021) 11:767026. doi: 10.3389/fonc.2021.767026 34868994 PMC8636012

[8] Zhao S Zhang X Gao F Chi H Zhang J Xia Z. Identification of copper metabolism-related subtypes and establishment of the prognostic model in ovarian cancer. Front Endocrinol (Lausanne). (2023) 14:1145797. doi: 10.3389/fendo.2023.1145797 36950684 PMC10025496

[9] Liu J Zhang P Yang F Jiang K Sun S Xia Z. Integrating single-cell analysis and machine learning to create glycosylation-based gene signature for prognostic prediction of uveal melanoma. Front Endocrinol (Lausanne). (2023) 14:1163046. doi: 10.3389/fendo.2023.1163046 37033251 PMC10076776

[10] Jin W Yang Q Chi H Wei K Zhang P Zhao G. Ensemble deep learning enhanced with self-attention for predicting immunotherapeutic responses to cancers. Front Immunol. (2022) 13:1025330. doi: 10.3389/fimmu.2022.1025330 36532083 PMC9751999

[11] Zhang Q He Y Luo N Patel SJ Han Y Gao R. Landscape and dynamics of single immune cells in hepatocellular carcinoma. Cell. (2019) 179:829–845.e820. doi: 10.1016/j.cell.2019.10.003 31675496

[12] Deng Y Shi M Yi L Naveed Khan M Xia Z Li X. Eliminating a barrier: Aiming at VISTA, reversing MDSC-mediated T cell suppression in the tumor microenvironment. Heliyon. (2024) 10:e37060. doi: 10.1016/j.heliyon.2024.e37060 39286218 PMC11402941

[13] Zhu H Zhao Y Wang Y Wei G Liu J. Understanding the relationship between cuproptosis and the development of hepatocellular carcinoma: implications for targeted therapies. Front Immunol. (2025) 16:1557223. doi: 10.3389/fimmu.2025.1557223 40145101 PMC11936877

[14] Huang Y Ge W Zhou J Gao B Qian X Wang W. The role of tumor associated macrophages in hepatocellular carcinoma. J Cancer. (2021) 12:1284–94. doi: 10.7150/jca.51346 PMC7847664 33531974

[15] Chi H Zhao S Yang J Gao X Peng G Zhang J. T-cell exhaustion signatures characterize the immune landscape and predict HCC prognosis via integrating single-cell RNA-seq and bulk RNA-sequencing. Front Immunol. (2023) 14:1137025. doi: 10.3389/fimmu.2023.1137025 37006257 PMC10050519

[16] Amann T Maegdefrau U Hartmann A Agaimy A Marienhagen J Weiss TS. GLUT1 expression is increased in hepatocellular carcinoma and promotes tumorigenesis. Am J Pathol. (2009) 174:1544–52. doi: 10.2353/ajpath.2009.080596 PMC2671384 19286567

[17] Kim YH Jeong DC Pak K Han ME Kim JY Liangwen L. SLC2A2 (GLUT2) as a novel prognostic factor for hepatocellular carcinoma. Oncotarget. (2017) 8:68381–92. doi: 10.18632/oncotarget.20266 PMC5620264 28978124

[18] Lis P Dyląg M Niedźwiecka K Ko YH Pedersen PL Goffeau A. The HK2 dependent “Warburg effect” and mitochondrial oxidative phosphorylation in cancer: targets for effective therapy with 3-bromopyruvate. Molecules. (2016) 21:1730. doi: 10.3390/molecules21121730 27983708 PMC6273842

[19] Yoo JJ Yu SJ Na J Kim K Cho YY Lee YB. Hexokinase-II inhibition synergistically augments the anti-tumor efficacy of sorafenib in hepatocellular carcinoma. Int J Mol Sci. (2019) 20:1292. doi: 10.3390/ijms20061292 30875800 PMC6471302

[20] Pajak B Siwiak E Sołtyka M Priebe A Zieliński R Fokt I. 2-deoxy-d-glucose and its analogs: from diagnostic to therapeutic agents. Int J Mol Sci. (2019) 21:234. doi: 10.3390/ijms21010234 31905745 PMC6982256

[21] Sun L Zhou F Shao Y Lv Z Li C. Sedoheptulose kinase bridges the pentose phosphate pathway and immune responses in pathogen-challenged sea cucumber Apostichopus japonicus. Dev Comp Immunol. (2020) 109:103694. doi: 10.1016/j.dci.2020.103694 32283109

[22] Lu DH Lv WW Li WX Gao YD. High PKM2 expression is independently correlated with decreased overall survival in hepatocellular carcinoma. Oncol Lett. (2018) 16:3603–10. doi: 10.3892/ol.2018.9100 PMC6096177 30127967

[23] Li SS Fitch WM Pan YC Sharief FS. Evolutionary relationships of vertebrate lactate dehydrogenase isozymes A4 (muscle), B4 (heart), and C4 (testis). J Biol Chem. (1983) 258:7029–32. doi: 10.1016/S0021-9258(18)32327-5 6853510

[24] Faloppi L Bianconi M Memeo R Casadei Gardini A Giampieri R Bittoni A. Lactate dehydrogenase in hepatocellular carcinoma: something old, something new. BioMed Res Int. (2016) 2016:7196280. doi: 10.1155/2016/7196280 27314036 PMC4903134

[25] Dang CV. MYC on the path to cancer. Cell. (2012) 149:22–35. doi: 10.1016/j.cell.2012.03.003 22464321 PMC3345192

[26] Zhang Z Deng X Liu Y Liu Y Sun L Chen F. PKM2, function and expression and regulation. Cell Biosci. (2019) 9:52. doi: 10.1186/s13578-019-0317-8 31391918 PMC6595688

[27] Wong KK Engelman JA Cantley LC. Targeting the PI3K signaling pathway in cancer. Curr Opin Genet Dev. (2010) 20:87–90. doi: 10.1016/j.gde.2009.11.002 20006486 PMC2822054

[28] Kohn AD Summers SA Birnbaum MJ Roth RA. Expression of a constitutively active Akt Ser/Thr kinase in 3T3-L1 adipocytes stimulates glucose uptake and glucose transporter 4 translocation. J Biol Chem. (1996) 271:31372–8. doi: 10.1074/jbc.271.49.31372 8940145

[29] Robey RB Hay N. Mitochondrial hexokinases, novel mediators of the antiapoptotic effects of growth factors and Akt. Oncogene. (2006) 25:4683–96. doi: 10.1038/sj.onc.1209595 16892082

[30] Zhang B Tang B Gao J Li J Kong L Qin L. A hypoxia-related signature for clinically predicting diagnosis, prognosis and immune microenvironment of hepatocellular carcinoma patients. J Transl Med. (2020) 18:342. doi: 10.1186/s12967-020-02492-9 32887635 PMC7487492

[31] Donne R Lujambio A. The liver cancer immune microenvironment: Therapeutic implications for hepatocellular carcinoma. Hepatology. (2023) 77:1773–96. doi: 10.1002/hep.32740 PMC9941399 35989535

[32] Xie H Xi X Lei T Liu H Xia Z. CD8(+) T cell exhaustion in the tumor microenvironment of breast cancer. Front Immunol. (2024) 15:1507283. doi: 10.3389/fimmu.2024.1507283 39717767 PMC11663851

[33] Wang D Zhang X Lu Y Wang X Zhu L. Hypoxia inducible factor 1α in hepatocellular carcinoma with cirrhosis: Association with prognosis. Pathol Res Pract. (2018) 214:1987–92. doi: 10.1016/j.prp.2018.09.007 30274686

[34] Chen H Chen J Yuan H Li X Li W. Hypoxia-inducible factor-1α: A critical target for inhibiting the metastasis of hepatocellular carcinoma. Oncol Lett. (2022) 24:284. doi: 10.3892/ol.2022.13404 35814827 PMC9260738

[35] Baltazar F Afonso J Costa M Granja S. Lactate beyond a waste metabolite: metabolic affairs and signaling in Malignancy. Front Oncol. (2020) 10:231. doi: 10.3389/fonc.2020.00231 32257942 PMC7093491

[36] Bantug GR Galluzzi L Kroemer G Hess C. The spectrum of T cell metabolism in health and disease. Nat Rev Immunol. (2018) 18:19–34. doi: 10.1038/nri.2017.99 28944771

[37] Zhang D Cui F Peng L Wang M Yang X Xia C. Establishing and validating an ADCP-related prognostic signature in pancreatic ductal adenocarcinoma. Aging (Albany NY). (2022) 14:6299–315. doi: 10.18632/aging.204221 PMC9417234 35963640

[38] Azoitei N Becher A Steinestel K Rouhi A Diepold K Genze F. PKM2 promotes tumor angiogenesis by regulating HIF-1alpha through NF-kappaB activation. Mol Cancer. (2016) 15:3. doi: 10.1186/s12943-015-0490-2 26739387 PMC4704385

[39] Palsson-McDermott EM Curtis AM Goel G Lauterbach MA Sheedy FJ Gleeson LE. Pyruvate kinase M2 regulates Hif-1alpha activity and IL-1beta induction and is a critical determinant of the warburg effect in LPS-activated macrophages. Cell Metab. (2015) 21:65–80. doi: 10.1016/j.cmet.2014.12.005 25565206 PMC5198835

[40] Jedlička M Feglarova T Janstova L Hortova-Kohoutkova M Frič J. Lactate from the tumor microenvironment-A key obstacle in NK cell-based immunotherapies. Front Immunol (2022) 13:932055. doi: 10.3389/fimmu.2022.932055 36330529 PMC9623302

[41] Jia W Liang S Cheng B Ling C. The role of cancer-associated fibroblasts in hepatocellular carcinoma and the value of traditional chinese medicine treatment. Front Oncol. (2021) 11:763519. doi: 10.3389/fonc.2021.763519 34868982 PMC8636329

[42] Zhengdong A Xiaoying X Shuhui F Rui L Zehui T Guanbin S. Identification of fatty acids synthesis and metabolism-related gene signature and prediction of prognostic model in hepatocellular carcinoma. Cancer Cell Int. (2024) 24:130. doi: 10.1186/s12935-024-03306-4 38584256 PMC11000322

[43] Li Q Wang C Wang Y Sun L Liu Z Wang L. HSCs-derived COMP drives hepatocellular carcinoma progression by activating MEK/ERK and PI3K/AKT signaling pathways. J Exp Clin Cancer Res. (2018) 37:231. doi: 10.1186/s13046-018-0908-y 30231922 PMC6146743

[44] Krammer J Digel M Ehehalt F Stremmel W Füllekrug J Ehehalt R. Overexpression of CD36 and acyl-CoA synthetases FATP2, FATP4 and ACSL1 increases fatty acid uptake in human hepatoma cells. Int J Med Sci. (2011) 8:599–614. doi: 10.7150/ijms.8.599 22022213 PMC3198256

[45] Nath A Li I Roberts LR Chan C. Elevated free fatty acid uptake via CD36 promotes epithelial-mesenchymal transition in hepatocellular carcinoma. Sci Rep. (2015) 5:14752. doi: 10.1038/srep14752 26424075 PMC4589791

[46] Soukupova J Malfettone A Bertran E Hernández-Alvarez MI Peñuelas-Haro I Dituri F. Epithelial-mesenchymal transition (EMT) induced by TGF-β in hepatocellular carcinoma cells reprograms lipid metabolism. Int J Mol Sci. (2021) 22:5543. doi: 10.3390/ijms22115543 34073989 PMC8197297

[47] Nakagawa H Hayata Y Kawamura S Yamada T Fujiwara N Koike K. Lipid metabolic reprogramming in hepatocellular carcinoma. Cancers (Basel). (2018) 10:447. doi: 10.3390/cancers10110447 30445800 PMC6265967

[48] Zu XY Zhang QH Liu JH Cao RX Zhong J Yi GH. ATP citrate lyase inhibitors as novel cancer therapeutic agents. Recent Pat Anticancer Drug Discov. (2012) 7:154–67. doi: 10.2174/157489212799972954 22339355

[49] Liu G Kuang S Cao R Wang J Peng Q Sun C. Sorafenib kills liver cancer cells by disrupting SCD1-mediated synthesis of monounsaturated fatty acids via the ATP-AMPK-mTOR-SREBP1 signaling pathway. FASEB J. (2019) 33:10089–103. doi: 10.1096/fj.201802619RR 31199678

[50] Montero J Morales A Llacuna L Lluis JM Terrones O Basañez G. Mitochondrial cholesterol contributes to chemotherapy resistance in hepatocellular carcinoma. Cancer Res. (2008) 68:5246–56. doi: 10.1158/0008-5472.CAN-07-6161 18593925

[51] Bai Y Yang J Cui Y Yao Y Wu F Liu C. Research progress of sirtuin4 in cancer. Front Oncol. (2020) 10:562950. doi: 10.3389/fonc.2020.562950 33585187 PMC7874138

[52] Li Z Li H Zhao ZB Zhu W Feng PP Zhu XW. SIRT4 silencing in tumor-associated macrophages promotes HCC development via PPARδ signalling-mediated alternative activation of macrophages. J Exp Clin Cancer Res. (2019) 38:469. doi: 10.1186/s13046-019-1456-9 31744516 PMC6862746

[53] Eguchi A Iwasa M. The role of elevated liver-type fatty acid-binding proteins in liver diseases. Pharm Res. (2021) 38:89–95. doi: 10.1007/s11095-021-02998-x 33534129

[54] Jin R Hao J Yi Y Sauter E Li B. Regulation of macrophage functions by FABP-mediated inflammatory and metabolic pathways. Biochim Biophys Acta Mol Cell Biol Lipids. (2021) 1866:158964. doi: 10.1016/j.bbalip.2021.158964 33984518 PMC8169605

[55] Neuschäfer-Rube F Schön T Kahnt I Püschel GP. LDL-dependent regulation of TNFα/PGE(2) induced COX-2/mPGES-1 expression in human macrophage cell lines. Inflammation. (2023) 46:893–911. doi: 10.1007/s10753-022-01778-y 36598592 PMC10188574

[56] Zhang X Yu C Zhao S Wang M Shang L Zhou J. The role of tumor-associated macrophages in hepatocellular carcinoma progression: A narrative review. Cancer Med. (2023) 12:22109–29. doi: 10.1002/cam4.v12.24 PMC10757104 38098217

[57] Hasan MN Capuk O Patel SM Sun D. The role of metabolic plasticity of tumor-associated macrophages in shaping the tumor microenvironment immunity. Cancers (Basel). (2022) 14:3331. doi: 10.3390/cancers14143331 35884391 PMC9316955

[58] Hicks KC Tyurina YY Kagan VE Gabrilovich DI. Myeloid cell-derived oxidized lipids and regulation of the tumor microenvironment. Cancer Res. (2022) 82:187–94. doi: 10.1158/0008-5472.CAN-21-3054 PMC8770601 34764204

[59] Zhang J Peng G Chi H Yang J Xie X Song G. CD8 + T-cell marker genes reveal different immune subtypes of oral lichen planus by integrating single-cell RNA-seq and bulk RNA-sequencing. BMC Health. (2023) 23:464. doi: 10.1186/s12903-023-03138-0 PMC10329325 37422617

[60] Chi H Gao X Xia Z Yu W Yin X Pan Y. FAM family gene prediction model reveals heterogeneity, stemness and immune microenvironment of UCEC. Front Mol Biosci. (2023) 10:1200335. doi: 10.3389/fmolb.2023.1200335 37275958 PMC10235772

[61] Chiu M Tardito S Pillozzi S Arcangeli A Armento A Uggeri J. Glutamine depletion by crisantaspase hinders the growth of human hepatocellular carcinoma xenografts. Br J Cancer. (2014) 111:1159–67. doi: 10.1038/bjc.2014.425 PMC4453854 25072259

[62] Jin L Alesi GN Kang S. Glutaminolysis as a target for cancer therapy. Oncogene. (2016) 35:3619–25. doi: 10.1038/onc.2015.447 PMC5225500 26592449

[63] Sun HW Yu XJ Wu WC Chen J Shi M Zheng L. GLUT1 and ASCT2 as predictors for prognosis of hepatocellular carcinoma. PLoS One. (2016) 11:e0168907. doi: 10.1371/journal.pone.0168907 28036362 PMC5201247

[64] Wang JB Erickson JW Fuji R Ramachandran S Gao P Dinavahi R. Targeting mitochondrial glutaminase activity inhibits oncogenic transformation. Cancer Cell. (2010) 18:207–19. doi: 10.1016/j.ccr.2010.08.009 PMC3078749 20832749

[65] Suzuki S Tanaka T Poyurovsky MV Nagano H Mayama T Ohkubo S. Phosphate-activated glutaminase (GLS2), a p53-inducible regulator of glutamine metabolism and reactive oxygen species. Proc Natl Acad Sci U S A. (2010) 107:7461–6. doi: 10.1073/pnas.1002459107 PMC2867754 20351271

[66] Xi J Sun Y Zhang M Fa Z Wan Y Min Z. GLS1 promotes proliferation in hepatocellular carcinoma cells via AKT/GSK3β/CyclinD1 pathway. Exp Cell Res. (2019) 381:1–9. doi: 10.1016/j.yexcr.2019.04.005 31054856

[67] Li B Cao Y Meng G Qian L Xu T Yan C. Targeting glutaminase 1 attenuates stemness properties in hepatocellular carcinoma by increasing reactive oxygen species and suppressing Wnt/beta-catenin pathway. EBioMedicine. (2019) 39:239–54. doi: 10.1016/j.ebiom.2018.11.063 PMC6355660 30555042

[68] Zhang J Wang C Chen M Cao J Zhong Y Chen L. Epigenetic silencing of glutaminase 2 in human liver and colon cancers. BMC Cancer. (2013) 13:601. doi: 10.1186/1471-2407-13-601 24330717 PMC3878668

[69] Liu J Zhang C Lin M Zhu W Liang Y Hong X. Glutaminase 2 negatively regulates the PI3K/AKT signaling and shows tumor suppression activity in human hepatocellular carcinoma. Oncotarget. (2014) 5:2635–47. doi: 10.18632/oncotarget.1862 PMC4058033 24797434

[70] Zhang X Zhang P Cong A Feng Y Chi H Xia Z. Unraveling molecular networks in thymic epithelial tumors: deciphering the unique signatures. Front Immunol. (2023) 14:1264325. doi: 10.3389/fimmu.2023.1264325 37849766 PMC10577431

[71] Gu J Wang Y Zhang H Gu H Zhu H. SIGLEC1 has the potential to be an immune-related prognostic indicator in colon adenocarcinoma: a study based on transcriptomic data and Mendelian randomization analysis. Discov Oncol. (2025) 16:324. doi: 10.1007/s12672-025-02093-2 40088346 PMC11910455

[72] Kuo TC Chen CK Hua KT Yu P Lee WJ Chen MW. Glutaminase 2 stabilizes Dicer to repress Snail and metastasis in hepatocellular carcinoma cells. Cancer Lett. (2016) 383:282–94. doi: 10.1016/j.canlet.2016.10.012 27725225

[73] Matos A Carvalho M Bicho M Ribeiro R. Arginine and arginases modulate metabolism, tumor microenvironment and prostate cancer progression. Nutrients. (2021) 13:4503. doi: 10.3390/nu13124503 34960055 PMC8704013

[74] Sun L Song L Wan Q Wu G Li X Wang Y. cMyc-mediated activation of serine biosynthesis pathway is critical for cancer progression under nutrient deprivation conditions. Cell Res. (2015) 25:429–44. doi: 10.1038/cr.2015.33 PMC4387561 25793315

[75] Chen J Cui L Lu S Xu S. Amino acid metabolism in tumor biology and therapy. Cell Death Dis. (2024) 15:42. doi: 10.1038/s41419-024-06435-w 38218942 PMC10787762

[76] Nong S Han X Xiang Y Qian Y Wei Y Zhang T. Metabolic reprogramming in cancer: Mechanisms and therapeutics. MedComm (2020). (2023) 4:e218. doi: 10.1002/mco2.v4.2 36994237 PMC10041388

[77] Siska PJ Jiao J Matos C Singer K Berger RS Dettmer K. Kynurenine induces T cell fat catabolism and has limited suppressive effects. vivo. EBioMedicine. (2021) 74:103734. doi: 10.1016/j.ebiom.2021.103734 34875457 PMC8652007

[78] Chen CT Wu PH Hu CC Nien HC Wang JT Sheu JC. Aberrant upregulation of indoleamine 2,3-dioxygenase 1 promotes proliferation and metastasis of hepatocellular carcinoma cells via coordinated activation of ahR and β-catenin signaling. Int J Mol Sci. (2021) 22:11661. doi: 10.3390/ijms222111661 34769098 PMC8583706

[79] Loftus RM Assmann N Kedia-Mehta N O’Brien KL Garcia A Gillespie C. Amino acid-dependent cMyc expression is essential for NK cell metabolic and functional responses in mice. Nat Commun. (2018) 9:2341. doi: 10.1038/s41467-018-04719-2 29904050 PMC6002377

[80] Wang Y Wang J Liu J Zhu H. Immune-related diagnostic markers for benign prostatic hyperplasia and their potential as drug targets. Front Immunol. (2024) 15:1516362. doi: 10.3389/fimmu.2024.1516362 39703506 PMC11655502

[81] Glasner A Levi A Enk J Isaacson B Viukov S Orlanski S. NKp46 receptor-mediated interferon-γ Production by natural killer cells increases fibronectin 1 to alter tumor architecture and control metastasis. Immunity. (2018) 48:107–119.e104. doi: 10.1016/j.immuni.2017.12.007 29329948

[82] Klausz K Pekar L Boje AS Gehlert CL Krohn S Gupta T. Multifunctional NK cell-Engaging antibodies targeting EGFR and NKp30 elicit efficient tumor cell killing and proinflammatory cytokine release. J Immunol. (2022) 209:1724–35. doi: 10.4049/jimmunol.2100970 36104113

[83] Xu Y Hao X Ren Y Xu Q Liu X Song S. Research progress of abnormal lactate metabolism and lactate modification in immunotherapy of hepatocellular carcinoma. Front Oncol. (2022) 12:1063423. doi: 10.3389/fonc.2022.1063423 36686771 PMC9853001

[84] Le Naour J Galluzzi L Zitvogel L Kroemer G Vacchelli EJO. Trial watch: IDO inhibitors in cancer therapy. Oncoimmunology (2020) 9:1777625. doi: 10.1080/2162402X.2020.1777625 32934882 PMC7466863

[85] Liu YX Feng JY Sun MM Liu BW Yang G Bu YN. Aspirin inhibits the proliferation of hepatoma cells through controlling GLUT1-mediated glucose metabolism. Acta Pharmacol Sin. (2019) 40:122–32. doi: 10.1038/s41401-018-0014-x PMC6318307 29925918

[86] Dai W Wang F Lu J Xia Y He L Chen K. By reducing hexokinase 2, resveratrol induces apoptosis in HCC cells addicted to aerobic glycolysis and inhibits tumor growth in mice. Oncotarget. (2015) 6:13703–17. doi: 10.18632/oncotarget.v6i15 PMC4537043 25938543

[87] Reyes RK Motiwala T Jacob ST. Regulation of glucose metabolism in hepatocarcinogenesis by microRNAs. Gene Expr. (2014) 16:85–92. doi: 10.3727/105221614X13919976902093 24801169 PMC4281965

[88] Jiang JX Gao S Pan YZ Yu C Sun CY. Overexpression of microRNA-125b sensitizes human hepatocellular carcinoma cells to 5-fluorouracil through inhibition of glycolysis by targeting hexokinase II. Mol Med Rep. (2014) 10:995–1002. doi: 10.3892/mmr.2014.2271 24865963

[89] Shang R Wang J Sun W Dai B Ruan B Zhang Z. RRAD inhibits aerobic glycolysis, invasion, and migration and is associated with poor prognosis in hepatocellular carcinoma. Tumour Biol. (2016) 37:5097–105. doi: 10.1007/s13277-015-4329-7 26546438

[90] Fan N Zhang X Zhao W Zhao J Luo D Sun Y. Covalent inhibition of pyruvate kinase M2 reprograms metabolic and inflammatory pathways in hepatic macrophages against non-alcoholic fatty liver disease. Int J Biol Sci. (2022) 18:5260–75. doi: 10.7150/ijbs.73890 PMC9461663 36147457

[91] Ma MKF Lau EYT Leung DHW Lo J Ho NPY Cheng LKW. Stearoyl-CoA desaturase regulates sorafenib resistance via modulation of ER stress-induced differentiation. J Hepatol. (2017) 67:979–90. doi: 10.1016/j.jhep.2017.06.015 28647567

[92] Rimassa L Finn RS Sangro B. Combination immunotherapy for hepatocellular carcinoma. J Hepatol. (2023) 79:506–15. doi: 10.1016/j.jhep.2023.03.003 36933770

[93] Tang H Liang Y Anders RA Taube JM Qiu X Mulgaonkar A. PD-L1 on host cells is essential for PD-L1 blockade-mediated tumor regression. J Clin Invest. (2018) 128:580–8. doi: 10.1172/JCI96061 PMC5785245 29337303

[94] Zhai X Zhang H Xia Z Liu M Du G Jiang Z. Oxytocin alleviates liver fibrosis via hepatic macrophages. JHEP Rep. (2024) 6:101032. doi: 10.1016/j.jhepr.2024.101032 38882603 PMC11177191

[95] Xiao J Lin H Liu B Xia Z Zhang J Jin J. Decreased S1P and SPHK2 are involved in pancreatic acinar cell injury. Biomark Med. (2019) 13:627–37. doi: 10.2217/bmm-2018-0404 31157539

[96] Xiao J Huang K Lin H Xia Z Zhang J Li D. Mogroside II(E) inhibits digestive enzymes via suppression of interleukin 9/interleukin 9 receptor signalling in acute pancreatitis. Front Pharmacol. (2020) 11:859. doi: 10.3389/fphar.2020.00859 32587518 PMC7298197

[97] Zhang H Xia T Xia Z Zhou H Li Z Wang W. KIF18A inactivates hepatic stellate cells and alleviates liver fibrosis through the TTC3/Akt/mTOR pathway. Cell Mol Life Sci. (2024) 81:96. doi: 10.1007/s00018-024-05114-5 38372748 PMC10876760

[98] Lu LG Zhou ZL Wang XY Liu BY Lu JY Liu S. PD-L1 blockade liberates intrinsic antitumourigenic properties of glycolytic macrophages in hepatocellular carcinoma. Gut. (2022) 71:2551–60. doi: 10.1136/gutjnl-2021-326350 PMC9664131 35173040

